# Generation and characterization of a monoclonal antibody against human BCL6 for immunohistochemical diagnosis

**DOI:** 10.1371/journal.pone.0216470

**Published:** 2019-05-07

**Authors:** Kunzhi Jia, Danping Zhang, Yanghai Wang, Yaju Liu, Xiangzhu Kong, Qinghai Yang, Huiling Chen, Chengjie Xie, Shihua Wang

**Affiliations:** 1 Key Laboratory of Pathogenic Fungi and Mycotoxins of Fujian Province, School of Life Sciences, Fujian Agriculture and Forestry University, Fuzhou, China; 2 Key Laboratory of Biopesticide and Chemical Biology of Education Ministry, School of Life Sciences, Fujian Agriculture and Forestry University, Fuzhou, China; 3 Fuzhou No.1 High School, Fuzhou, China; 4 Fuzhou Maixin Biotech. Co., Ltd, Fuzhou, China; National Institutes of Health, UNITED STATES

## Abstract

**Background:**

Human B-cell lymphoma 6 (BCL6) gene, usually coding protein of 706 amino acids, is closely associated with large B cell lymphoma. Researches showed that protein mutation or change of expression levels usually happened in the mounting non-hodgkin lymphoma (NHL). Thus BCL6 is considered to be involved in germinal center (GC)-derived lymphoma.

**Results:**

The BCL6_1-350_ gene codons were optimized for prokaryotic system. After expression of BCL6_1-350_ in *E*. *coli*, the BCL6_1-350_ protein was purified with Ni column. Then the BCL6_1-350_ protein, mixing with QuickAntibody-Mouse5W adjuvant, was injected into Balb/c mice. After immunization and cell fusion, a stable cell line named 1E6A4, which can secrete anti-BCL6 antibody, was obtained. The isotype of 1E6A4 mAb was determined as IgG_2a_, and the affinity constant reached 5.12×10^10^ L/mol. Furthermore, the specificity of the mAb was determined with ELISA, western blot and immunohistochemistry. Results indicated that the 1E6A4 mAb was able to detect BCL6 specifically and sensitively.

**Conclusions:**

BCL6_1-350_ antigen has been successfully generated with an effective and feasible method, and a highly specific antibody named 1E6A4 against BCL6 has been screened and characterized in this study, which was valuable in clinical diagnosis.

## Introduction

Human B-cell lymphoma 6 (BCL6) gene, usually coding protein of 706 amino acids, is closely associated with large B cell lymphoma. Researches showed that protein mutation or change of expression levels usually happened in the mounting non-hodgkin lymphoma (NHL). Taking classical diffusing large B cell lymphoma (DLBCL) for example, Ye et al found about 33% DLBCL contains truncated BCL6 gene within its 5’ noncoding region, leading to decreased expression levels of BCL6 protein [1]. Further study showed that BCL6 plays a role in transcription repression and anti-apoptosis, as a transcriptional repressor with zinc finger and POZ domain [2]. BCL6 is mainly expressed in germinal center (GC) B and T cells [3], and can repress IL-4 mediated intracellular gene expression [4]. Experimental evidence showed that BCL6 was essential during GC formation [3]. On the other hand, the alteration and deregulation of BCL6 were usually found in B-Cell lymphomas and GC-derived lymphomas (follicular lymphomas). In a word, mounting evidence showed that BCL6 played an important role in the occurrence and development of GC-derived lymphoma.

With the deepening of research about BCL6, researchers found that the abnormality of BCL6 expression could be used as the diagnosis marker of GC-derived lymphomas. For example, Seeqmiller et al found the expression of BCL6 was an effective marker of Burkitt lymphoma, and was significant for prognostic survival [5]. Herbeck et al found that BCL6 was significantly helpful for diagnosis of Hodgkin lymphoma [6]. The expression of BCL6 varied in DLBCL, which was useful for subsequent therapy [7]. Interestingly, the expression of BCL6 is sometimes selected as the therapy target depending on the detailed information of BCL6 expression. Cardenas et al found that the inhibitors of BCL6 could effectively remedy DLBCL with BCL6 positive expression [8,9]. Therefore, it is valuable to develop antibody against BCL6 for clinical application. The antibody recognizing various epitopes of BCL6 offers the possible opportunities for mechanism study and clinical application of lymphomas.

Considering the various modifications in human proteins, eukaryotic system is usually used for generating immune protein antigen for antibodies in immunohistochemistry (IHC). Compared with the time-consuming and technique-requiring method to express BCL6 in a eukaryotic cell line, a method for BCL6 expression in prokaryotic system was developed in this study. Hence, an effective, reliable and feasible method was established to prepare an anti-BCL6 mAb by expressing BCL6_1-350_ in *E*. *coli*. The prepared mAb against BCL6 from prokaryotic system can be used in western blot, ELISA, and IHC diagnosis based on its high specificity in this study. Thus, this methodology will provide a reference for effectively generating IHC antibodies using prokaryotic system, and the antibody against BCL6 will be valuable for BCL6-oriented study and clinical applications.

## Materials and methods

### Ethics approval and consent to participate

All animal experiments obeyed the protocols approved by the Animal Ethics Committee of the Fujian Agriculture and Forestry University. Not applicable for consent to participate.

### Materials and chemicals

The anti-His_6_ antibody and HRP-labeled goat anti-mouse IgG were purchased from ZSGB-Bio Co., Ltd (Beijing, China). The control antibody LN22 (Newcastle, United Kingdom) and MaxVision/HRP IHC kit were provided by Fuzhou Maixin Biotech Co., Ltd (Fuzhou, China). SP2/0 myeloma cells were stored in our laboratory. Hypoxanthine, aminopterin and thymidine supplement (HAT), hypoxanthine and thymidine supplement (HT) were purchased from Sigma-Aldrich (Shanghai, China). RPMI 1640 medium powder and fetal bovine serum (FBS) were purchased from Thermo Fisher Scientific (Shanghai, China). Balb/c mice were from Wushi Animal Laboratory (Shanghai, China). All animal studies were approved by the Animal Ethics Committee of the Fujian Agriculture and Forestry University and have been carried out in accordance with the NIH guidelines for the care and use of Laboratory animals.

### Cell culture

SP2/0 cells were cultured in RPMI-1640 medium with 10% FBS. Hybridoma cells were cultured in RPMI-1640 medium with 20% FBS containing HAT. After 10 days, HAT in RPMI-1640 medium was replaced with HT. Then a week later, the hybridoma cells were cultured in RPMI-1640 medium with 10% FBS [10].

### Codon optimization and synthesis of BCL6_1-350_ gene

The coding region for BCL6_1-350_ (GenPept, NP_001124317.1) was optimized for expression in *E*. *coli* BL21 (DE3) and evaluated by graphical codon usage analyser (http://gcua.schoedl.de) [10]. The optimized coding DNA for BCL6_1-350_ was synthesized by Nanjing Genscript Biotech Co., Ltd (Nanjing, China).

### Expression and identification of the BCL6_1-350_ protein

The optimized gene for BCL6_1-350_ was constructed into pET-28a and expressed in *E*. *coli* BL21 (DE3). Then the expressed recombinant protein His-BCL6_1-350_ was purified by affinity chromatography purification. The concentrations of His-BCL6_1-350_ protein were measured with BCA methods, and then BCL6_1-350_ protein was identified by western blot.

### Animal immunization, cell fusion, and hybridoma screening

The 6- to 8-week-old female mice were immunized intramuscularly using 20 μg BCL6_1-350_ mixed with the adjuvant of QuickAntibody-Mouse5W. After three times immunization, the titers of tail blood from the immunized mice were tested with iELISA. The mice with high titer were injected intraperitoneally (i.p.) as the further immunization with 20 μg BCL6 in 100 μL 0.9% saline solution. Three days later, splenocytes were fused with SP2/0 cells at a ratio of 10: 1 with PEG1450. Then the fused cells were distributed into 96-well microplates and cultured in RPMI 1640 with 20% FBS/HAT medium. Five days later, half medium was substituted with fresh medium. Ten days later, positive hybridoma cells were determined by iELISA. The positive clones with high titer were chosen for further sub-cloning, until the positive percentage was up to 100% [10,11].

### Characterization of the positive hybridoma cells

The isotype of 1E6A4 mAb was determined with mouse monoclonal antibody isotyping (IgA, IgM, IgG_1_, IgG_2a_, IgG_2b_, IgG_3_) kit. Chromosome analysis was carried out as described previously [12]. In brief, the hybridoma cells were stained with Giemsa stain solution, and the chromosome number was counted under the fluorescence microscope.

### Production of anti-BCL6 mAb and titer determination

Each Balb/c mouse was injected i.p. with 0.5 mL paraffin oil. Seven days later, approximately 1×10^6^ positive hybridoma cells were injected into the mouse abdominal cavity. After one week, the ascites fluid was collected by the needle and centrifuged at 12000 r/min for 20 min. The supernatant was absorbed and stored in -20°C fridge. The anti-BCL6 mAb was purified with Protein G and analyzed with 10% SDS-PAGE. The concentration of the purified mAb was determined with BCA protein assay [10]. Titer determination of 1E6A4 mAb was determined with iELISA. BCL6_1-350_ antigens were coated at the concentration of 1.7 μg/mL, and the 1E6A4 mAb were diluted from the original concentration of 715 ng/mL.

### Determination of affinity and specificity of anti-BCL6 antibody

The affinity of antibody against BCL6 was determined with iELISA as described previously [10]. Various concentrations of BCL6 proteins (425.00, 106.25, and 26.56 ng/mL) were used as coating antigens. Relative affinity of anti-BCL6 mAb was measured by determining the 50% inhibition of control values (IC_50_). The affinity constant (K_aff_) was calculated by using the reported method. The specificity of mAb against BCL6 was determined by two kinds of methods (iELISA, western blot). For iELISA, different human proteins (BCL6, programmed cell death 1 ligand 1 (PD1-L1), P-glycoprotein (PGY) and podoplanin (PDPN)), which are presently available in our lab, were used as random control in this study. For western blot, BCL6 was resolved by 10% SDS-PAGE gel, and transferred into PVDF membrane. The anti-BCL6 mAb as primary antibody was diluted 1: 10000 with 5% PBSM to incubate with the PVDF membrane for 1 h. The pictures were taken by fully functional multicolor fluorescence imaging instrument [13].

### Determination of the epitope of BCL6 for 1E6A4 mAb

The optimized BCL6_1-350_ gene was divided into three gene fragments, which has the suitable size for expression analysis. Each gene fragment contains 117 amino acid coding codons except the third fragment, which has 116 amino acid coding codons. BCL6 gene fragments were synthesized by General Biosystems (Chuzhou, China) and cloned into pET-28a plasmid. These fragments fused with His_6_ tags were expressed in *E*.*coli* BL21 (DE3). The protein expression was analyzed with SDS-PAGE, and the epitope for 1E6A4 was determined with western blot.

### IHC tests of mAb

IHC experiments were approved by Ethics Committee of Fuzhou University. Tissue samples are human samples from hospital without the consents of patients (the data were analyzed anonymously). The IHC was performed with the protocol described before [10]. In brief, after being deparaffinized in xylene and rehydrated in graded alcohol, the formalin-fixed paraffin-embedded specimens were treated with boiling citrate buffer (10 mmol/L, pH 6.0) for 10 min using pressure cooker. Anti-BCL6 mAb (1: 100 for 1E6A4, 1: 75 for LN22 as positive control) was added into the section and incubated at room temperature for 1 h. After final washing, the solution of MaxVision/HRP-Polymer anti-mouse IHC kit was dropped into the slice and incubated for 25 min. The DAB chromogenic liquid was added into the section for 10 min, and then stopped with water. Finally, the section was sealed by neutral gum and observed under microscope.

## Results

### Cloning and expression of BCL6_1-350_ gene in prokaryotic system

**Fig 1:**
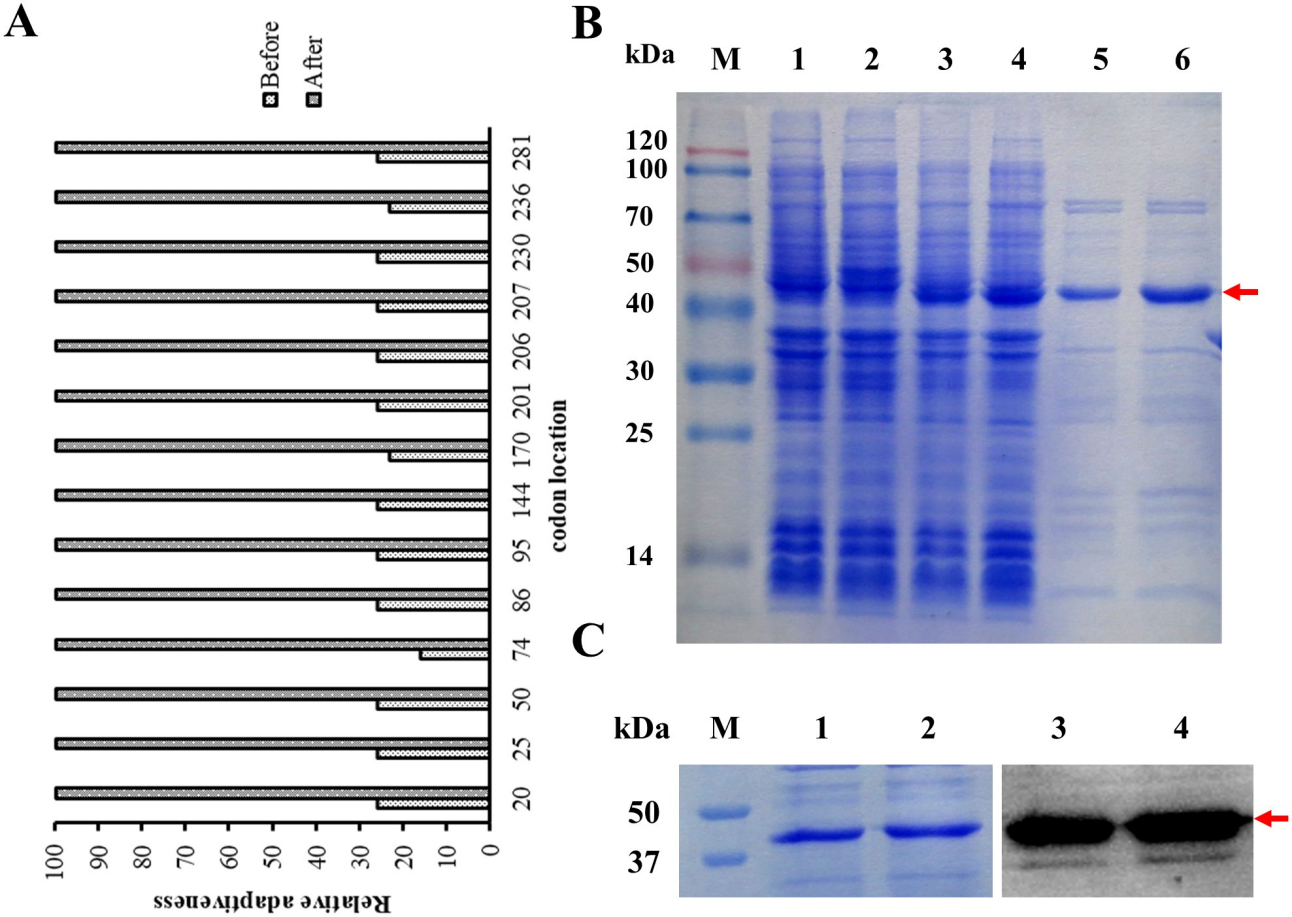
Cloning and expression of BCL6_1-350_ in prokaryotic system. (A) The relative adaptiveness comparison of representative BCL6_1-350_ codons between original and optimized sequence in *E*.*coli*. The relative adaptiveness analysis of BCL6_1-350_ codons for original and optimized sequence was compared in S1 Fig. 14 codons of BCL6_1-350_ were below 30% of relative adaptiveness, which were chosen for the comparison with the optimized codons of BCL6_1-350_. (B) Expression and Purification of BCL6_1-350_. *E*.*coli* containing indicated vector were cultured in LB medium at 37°C. 3h further culture with IPTG induction or control was conducted for protein expression, when OD of *E*.*coli* reached 0.6 ~ 0.8. Then the expression profiles of *E*.*coli* were analyzed with SDS-PAGE analysis. M: Marker (Blue plus II protein marker); lane 1: expression profile of *E*.*coli* containing control vector; lane 2: expression profile for BCL6_1-350_ expression vector without induction; lane 3–4: the duplicate expression profile for BCL6_1-350_ vector after IPTG induction, lane 5–6: the duplicate of BCL6_1-350_ after purification. (C) Western blot result of BCL6_1-350_. M: Marker (Precision Plus Protein Dual color standards). The purified BCL61-350 protein was resolved by 10% SDS-PAGE, and then transferred into PVDF membrane for immunoblot with anti-His_6_ antibody. Lane 1–2: the duplicate for SDS-PAGE of BCL6_1-350_ protein, lane 3–4: the duplicate for western blot result of BCL6_1-350_ reacted with anti-His_6_ tag monoclonal antibody.

To express BCL6_1-350_ gene in prokaryotic system, the codon sequence of BCL6_1-350_ was optimized (S1 Table). The optimized codons were analyzed with the graphical codon usage analyser (http://gcua.schoedl.de). As shown in Fig 1A, the relative adaptivenesses of codons, which were less than 30% in BCL6_1-350_ before optimization, were all optimized to 100%, indicating that the codons could be well expressed in *E*. *coli*. Then the optimized gene sequence was synthesized and cloned into the vector pET-28a. As shown in Fig 1B, BCL6_**1-350**_ gene was well expressed after IPTG induction with the expected size and purified from *E*. *coli* BL21 (DE3), indicating that the strategy of optimization and expression in prokaryotic system for BCL6_**1-350**_ gene is successful. The purified BCL6_**1-350**_ antigen was confirmed with western blot analysis. As in Fig 1C, the purified BCL6_1-350_ was well recognized by anti-his_6_ antibody.

### Screening and characterization of hybridoma cells secreting antibody against BCL6

**Fig 2:**
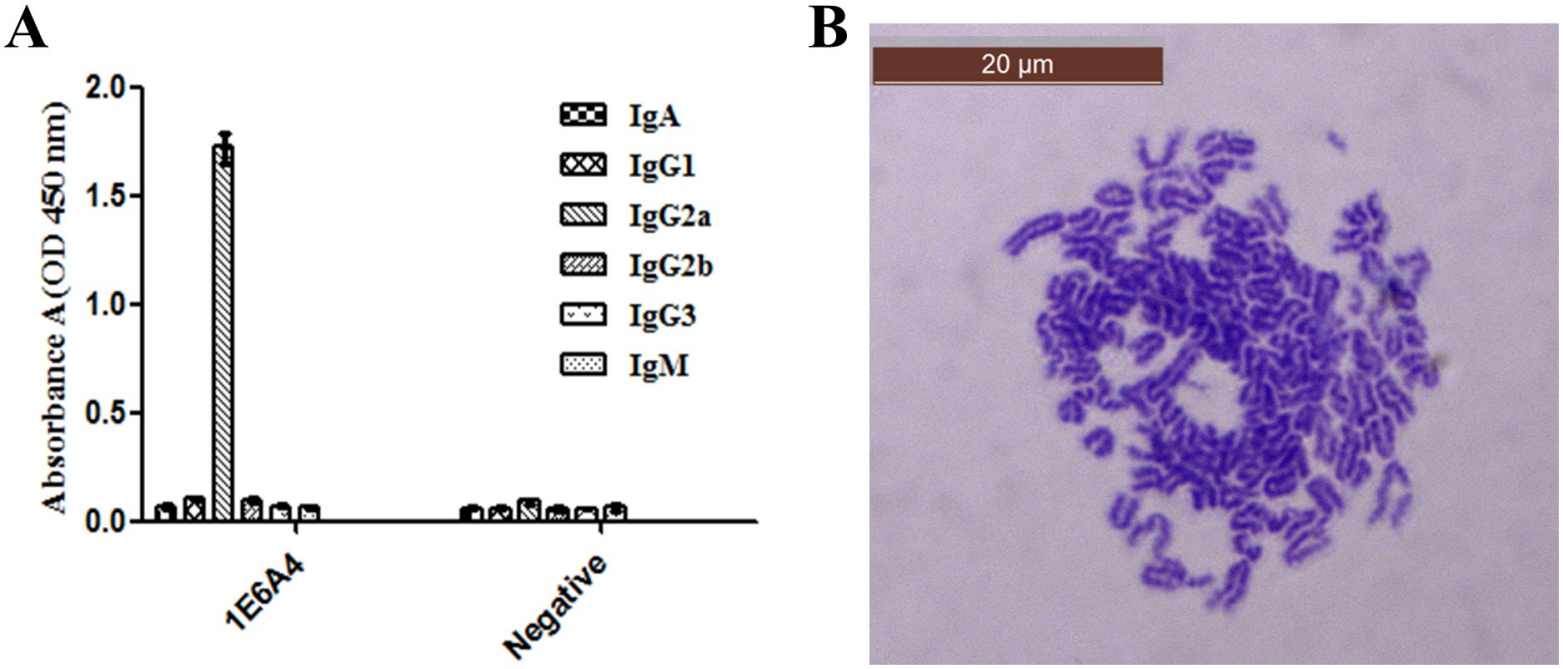
Characterization of positive hybridoma clone. (A) The isotype result of monoclonal antibody from 1E6A4 hybridoma cells. The experiment of antibody isotyping was conducted with isotyping kit, and the isotype of 1E6A4 is IgG2a. (B) Chromosome analysis of 1E6A4 hybridoma cell (10×100). The chromosome analysis of 1E6A4 cell line is 102±6.

To get B cells secreting anti-BCL6 antibodies, Balb/c mice were immunized with BCL6_1-350_. Then spleen cells from the mouse, which has high titer of antiserum against BCL6, were isolated and fused to SP2/0 myeloma cells. After cell hybridization, a cell line, 1E6A4, stably secreting anti-BCL6 antibodies was screened out. The isotype of antibody from 1E6A4 was determined with antibody isotyping kit. As shown in Fig 2A, the isotype of antibody 1E6A4 was IgG2a. Giemsa staining was used to determine the chromosome number of 1E6A4 cells. As shown in Fig 2B, 1E6A4 cells have 102±6 chromosomes, approximately equal to the total chromosome number of spleen cell (39±1) and SP2/0 myeloma cell (66±4). All these results demonstrated that the 1E6A4 cell line was successfully fused from spleen cell and SP2/0 myeloma cell, and qualified for further application.

### Purification of anti-BCL6 monoclonal antibody

**Fig 3:**
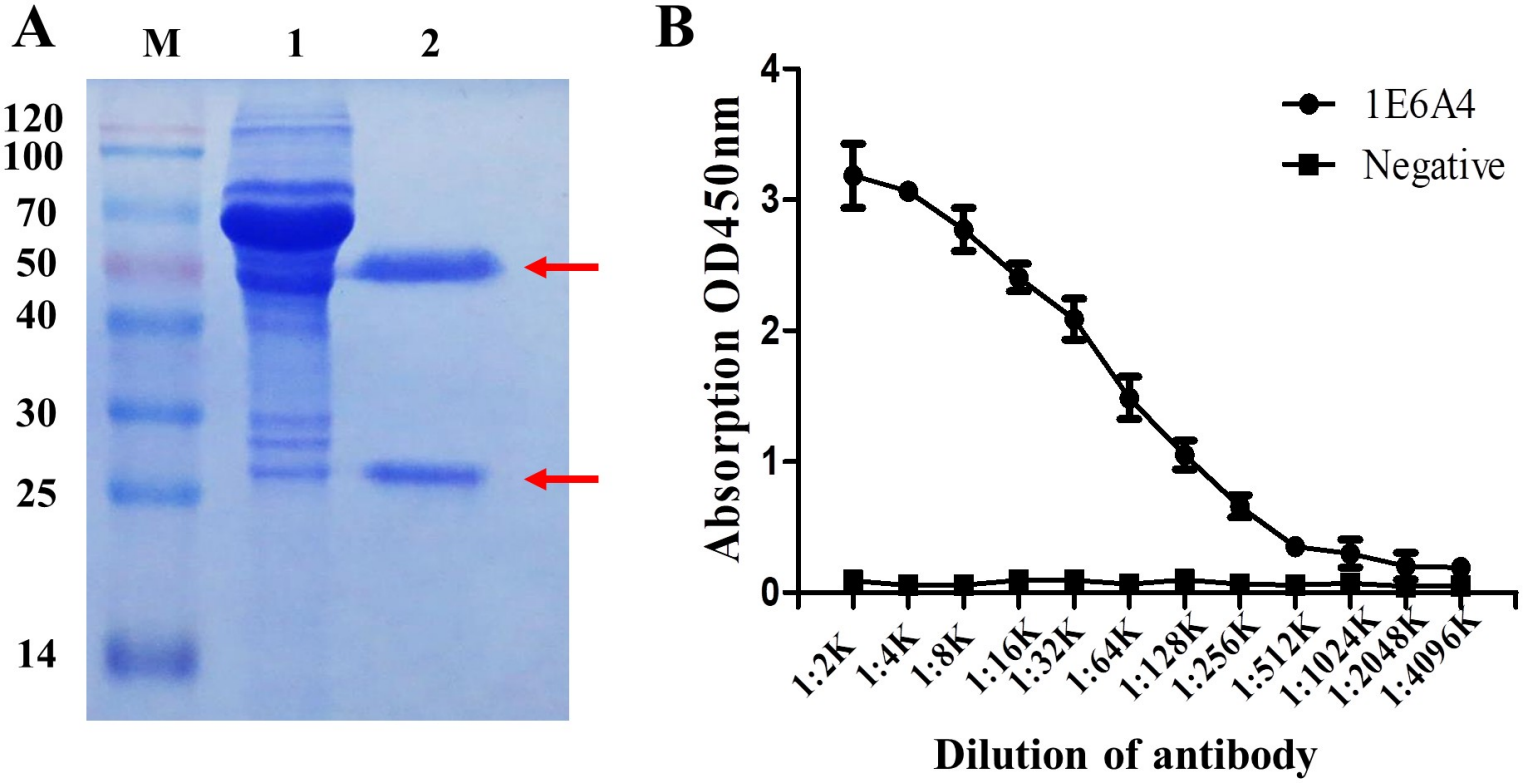
Purification of antibody against BCL6. (A) SDS-PAGE analysis of antibodies against BCL6 after purification. M: Marker (Blue plus II protein marker); lane 1: protein profile of ascites fluid; lane 2: the purified mAb against BCL6 (1E6A4). The bands shown with arrows indicate heavy and light chains respectively from 1E6A4 antibodies. (B) Determiantion of the 1E6A4 antibody titer with iELISA. BCL6_1-350_ antigens were coated, and antibodies were diluted as shown.

To get enough antibodies, the 1E6A4 hybridoma cells were injected i.p. into the peritoneal cavity of pristine-primed Balb/c mice. A week later, the mice ascites fluids were collected and then used for antibody purification. As shown in Fig 3A, two distinct bands, consistent with heavy chain and light chain of antibody, were observed, indicating that 1E6A4 mAb were successfully purified by Protein G. Then the antibody titer against BCL6 was determined with iELISA. As shown in Fig 3B, the 1E6A4 titer reached up to 2.05×10^6^, demonstrating that the anti-BCL6 1E6A4 mAb was suitable for further characterization.

### Specificity and affinity of anti-BCL6 monoclonal antibody

**Fig 4:**
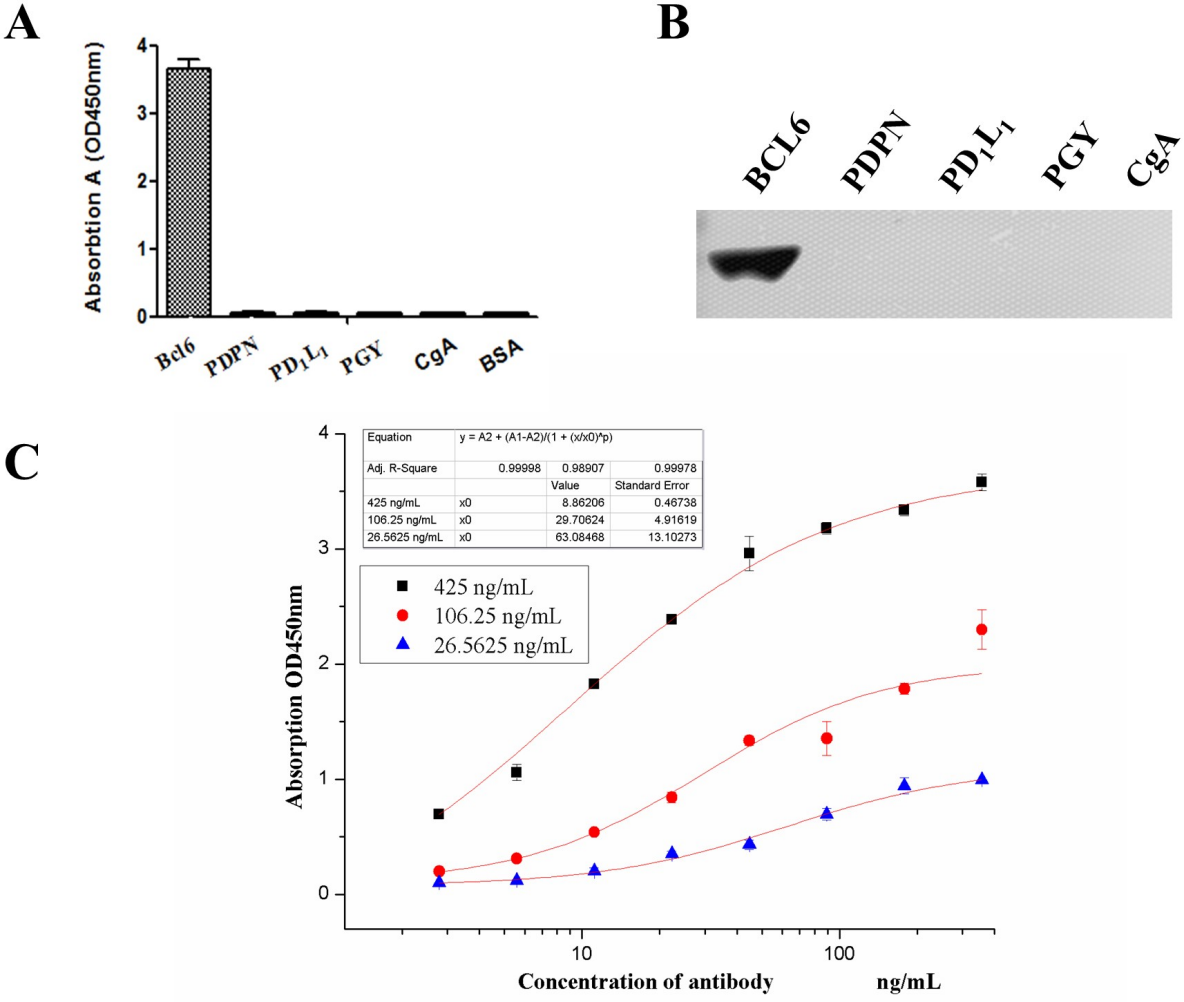
Characterization of 1E6A4 mAb. (A) The specificity of the 1E6A4 mAb was determined by indirect ELISA (BCL6: B-cell lymphoma 6 protein; PDPN: Podoplanin; PD1-L1: Programmed cell death 1 ligand 1; PGY: P-glycoprotein; CgA: Chromogranin A; BSA: Bovine serum albumin). (B) The specificity of the 1E6A4 mAb was determined by Western Blot. (C) Affinity of 1E6A4 mAb was analyzed by iELISA. Different concentrations (26.56, 106.25, and 425.00 ng/mL) of coating antigen (BCL6) were used. The affinity constant is determined as 5.12×10^10^ L/mol.

To evaluate the value of 1E6A4 mAb, the affinity and specificity of antibodies were determined with iELISA. As shown in Fig 4A, the 1E6A4 mAb was sensitively responsive to BCL6, but not cross-reactive to random control proteins, such as PDPN, PD1-L1, PGY and CgA, indicating the 1E6A4 antibodies have specificity for BCL6. The specificity of 1E6A4 antibodies was also evaluated with western blot. As shown in Fig 4B, the 1E6A4 mAb specifically recognized BCL6 rather than control proteins. Then the affinity analysis of 1E6A4 mAb was performed, and the result showed that the affinity was up to 5.12×10^10^ L/mol (Fig 4C), indicating that the 1E6A4 mAb was potentially valuable for further application in diagnosis [14,15].

### Determination of the epitope for anti-BCL6 monoclonal antibody

**Fig 5:**
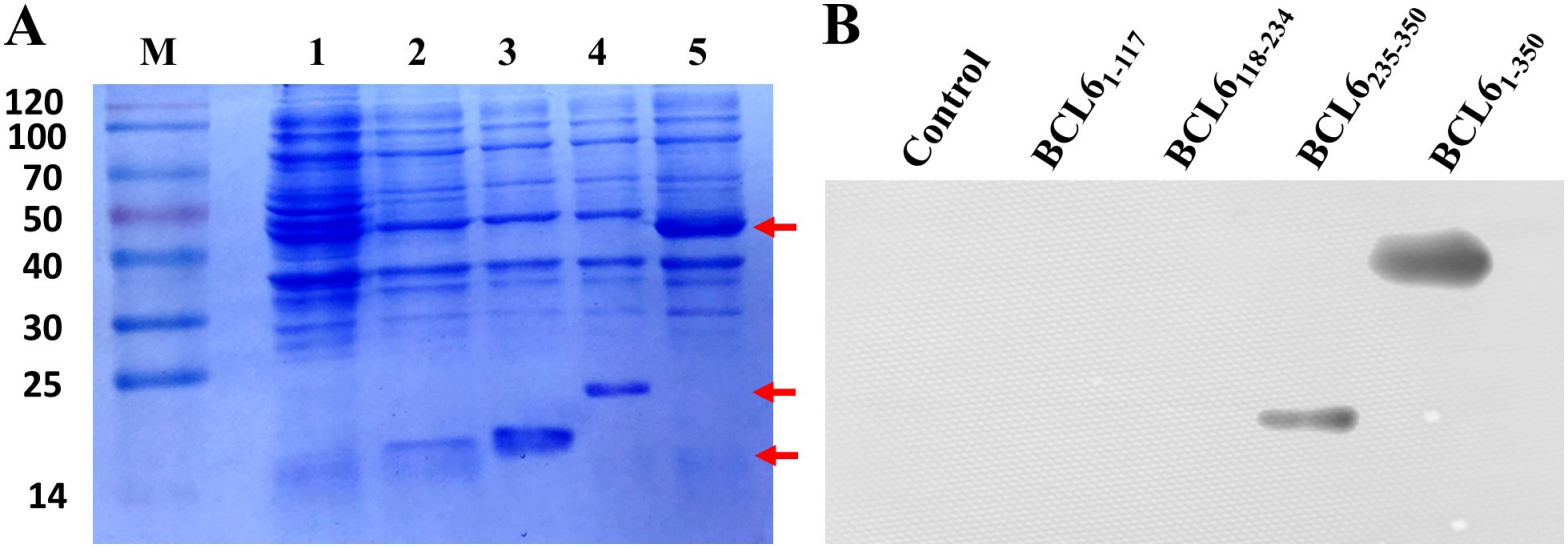
The epitope analysis of BCL6 for 1E6A4 mAb. (A) SDS-PAGE analysis of BCL6_1-350_ fragments expression in *E*.*coli*. M: Marker (Blue plus II protein marker); lane 1: Control; lane 2: BCL6_1-117_; lane 3: BCL6_118-234_; lane 4: BCL6_235-350_; lane 5: BCL6_1-350_. (B) The epitope of BCL6 for 1E6A4 mAb was determined by western blot.

To determine the epitope of BCL6 for 1E6A4 antibody, BCL6_1-350_ gene was divided into three fragments, each consisting of about 117 amino acids. These fragments were fused expressed with His_6_ tag in pET-28a as BCL6_1-350_. As expected, BCL6 fragments were well expressed in *E*.*coli* (Fig 5A). The first two fragments exhibited ~16 kD, the theory molecular size. Interesting, the third fragment exhibited ~ 24 kD, about 8 kD larger than the theory size (Fig 5A). At the same time, BCL6_1-350_ also exhibited the similar electrophoresis phenomenon (Fig 5A). Then the epitope for 1E6A4 mAb was further determined with western blot method. As shown in Fig 5B, the third fragment (BCL6_235-350_), as well as BCL6_1-350_, was recognized by 1E6A4 antibody, demonstrating the BCL6_235-350_ fragment was the epitope of BCL6 for 1E6A4 antibody.

### Application of anti-BCL6 antibody in IHC

**Fig 6:**
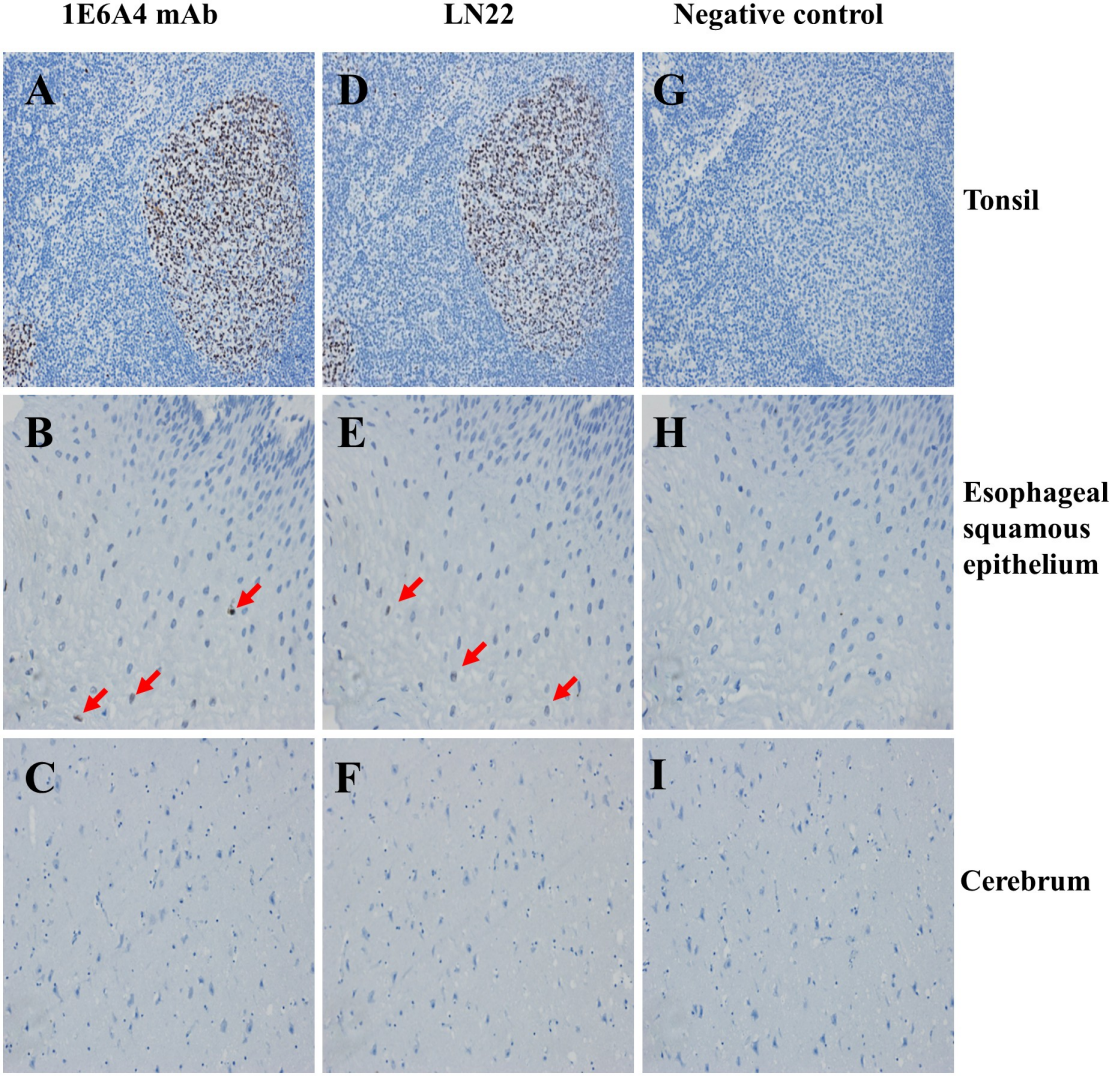
The immunohistochemistry tests of 1E6A4 mAb in difference tissues. The immunohistochemistry tests of 1E6A4 mAb was determined in tonsil (A) (×200), esophageal squamous epithelium (B)(×400), and cerebrum (C) (×200). The images (D), (E) and (F) showed the staining of LN22 antibody for corresponding tissues, as positive control. The images of (G), (H) and (I) showed the staining of IgG control antibody for corresponding tissues, as negative control. The cells reactive with antibodies were stained with brown color. Some stained cells were indicated with arrows.

Considering the significance of BCL6 detection in clinical diagnosis, the 1E6A4 antibodies were tested with IHC assay in different tissue samples. As shown in Fig 6, the 1E6A4 mAb is able to sensitively detect BCL6 (staining with brown color), equal to the positive control group (LN22). Thus 1E6A4 mAb can be used to specifically recognize BCL6 in tonsil and esophageal squamous epithelium. The stained cells indicated that BCL6 expressing cells were located in these tissues, which were valuable in clinical practice [16]. As control tissue, no cell was stained in cerebrum as expected (Fig 6). All these results showed the 1E6A4 mAb had good potential value to detect BCL6 expressing tumor cells in clinic.

## Discussion

As a eukaryotic protein, perfect BCL6 antigen for antibody generation should be from eukaryotic system. In this study, to simplify the antigen preparation, BCL6_1-350_ gene fragment was selected and optimized for expression in *E*. *coli*. The codons of BCL6, which have the least relative adaptiveness (less than 30%) in *E*. *coli*, have been optimized to be 100% adaptiveness after optimization (Fig 1A). At the same time, the observed high expression levels also indicated that the optimization strategy and expression in prokaryotic system for BCL6 is successful (Fig 1B).

Meanwhile, a valuable hybridoma secreting antibodies against BCL6 was successfully screened using the antigen from prokaryotic system in this study. This antibody, 1E6A4, exhibited high specificity and affinity for BCL6 (Fig 4). At the same time, the affinity of 1E6A4 mAb was high up to 5.12×10^10^ L/mol (Fig 4C). This affinity is far beyond the baseline (10^7^ L/mol) required for further applications [17], suggesting that the 1E6A4 mAb is potentially valuable in clinical application.

Although eukaryotic proteins usually undergo various post-translational modifications, BCL6 specific antibody applicable in IHC has been successfully generated using antigen from prokaryotic system in this study. This methodology of generating antigen from prokaryotic system is easy and less time-consuming. As known, epitopes have special significance for antibody generation and clinical diagnosis. To better make use of the 1E6A4 antibody, the epitope of BCL6 for antibody has been characterized (Fig 5B). The 1E6A4 antibodies, recognizing the epitope BCL6_235-350_, will be useful for the detection of BCL6, which is significant in diagnosis of DLBCL or other GC-derived lymphoma [15,16,18]. Although other antibodies against BCL6, such as LN22, can also be used for diagnosis purpose, 1E6A4 can have better exhibition and give more useful information, considering its detailed characterization described above.

Interestingly, BCL6_235-350_ consisting of many charged amino acids exhibits ~8 kDa lag in electrophoresis (Fig 5A), leading to the lag effect of BCL6_1-350_ in electrophoresis compared with the theory molecular weight (Figs 1B & 5A). It is to be noted that BCL6_235-350_ also is the epitope for 1E6A4, suggesting that charged amino acids tend to be the components of antibodies useful in IHC [10].

Our study also indicated that the 1E6A4 mAb is qualified for clinical application with IHC experiments. As shown in Fig 6, the 1E6A4 mAb could sensitively detect BCL6 in tonsil and esophageal squamous epithelium samples, which is equal to the LN22 control antibody, a commercial antibody applicable in IHC diagnosis [14,15]. This suggested that 1E6A4 is valuable in further clinical application. Although our results were shown just from tonsil, esophageal squamous epithelium, and cerebrum, it is reasonable that the 1E6A4 is also applicable in other tissues.

## Conclusions

In summary, we have successfully generated BCL6_1-350_ antigen with an effective and feasible method, which is obviously suitable for preparing a BCL6 mAb. Moreover, a highly specific antibody named 1E6A4 against BCL6 has been screened and characterized in this study, which was valuable in clinical diagnosis. At the same time, this 1E6A4 antibody recognizing BCL6_235-350_ will be useful in BCL6-oriented study and clinical practice, considering the potentially complicated regulation and presence of BCL6 in cell events and various diseases.

## Supporting information

S1 Table BCL6_1-350_ coding DNA sequence. (DOC)

S1 Fig Comparison of BCL6_1-350_ relative adaptiveness between original (before) and optimized sequence (after). (DOC)
