# Supplementary material for: Generation and characterization of a monoclonal antibody against human BCL6 for immunohistochemical diagnosis
Source: PLoS One. 2019 May 7;14(5):e0216470. doi: 10.1371/journal.pone.0216470 (PMC6504089; doi:10.1371/journal.pone.0216470)
Supplement: S1 Table — (DOC) [file pone.0216470.s001.doc]

**S1 Table. BCL6_1-350_ Coding DNA Sequence.**

| **BCL6_1-350_ optimization** | **Coding DNA Sequence** |
| --- | --- |
| Before | ATGGCCTCGCCGGCTGACAGCTGTATCCAGTTCACCCGCCATGCCAGTGATGTTCTTCTCAACCTTAATCGTCTCCGGAGTCGAGACATCTTGACTGATGTTGTCATTGTTGTGAGCCGTGAGCAGTTTAGAGCCCATAAAACGGTCCTCATGGCCTGCAGTGGCCTGTTCTATAGCATCTTTACAGACCAGTTGAAATGCAACCTTAGTGTGATCAATCTAGATCCTGAGATCAACCCTGAGGGATTCTGCATCCTCCTGGACTTCATGTACACATCTCGGCTCAATTTGCGGGAGGGCAACATCATGGCTGTGATGGCCACGGCTATGTACCTGCAGATGGAGCATGTTGTGGACACTTGCCGGAAGTTTATTAAGGCCAGTGAAGCAGAGATGGTTTCTGCCATCAAGCCTCCTCGTGAAGAGTTCCTCAACAGCCGGATGCTGATGCCCCAAGACATCATGGCCTATCGGGGTCGTGAGGTGGTGGAGAACAACCTGCCACTGAGGAGCGCCCCTGGGTGTGAGAGCAGAGCCTTTGCCCCCAGCCTGTACAGTGGCCTGTCCACACCGCCAGCCTCTTATTCCATGTACAGCCACCTCCCTGTCAGCAGCCTCCTCTTCTCCGATGAGGAGTTTCGGGATGTCCGGATGCCTGTGGCCAACCCCTTCCCCAAGGAGCGGGCACTCCCATGTGATAGTGCCAGGCCAGTCCCTGGTGAGTACAGCCGGCCGACTTTGGAGGTGTCCCCCAATGTGTGCCACAGCAATATCTATTCACCCAAGGAAACAATCCCAGAAGAGGCACGAAGTGATATGCACTACAGTGTGGCTGAGGGCCTCAAACCTGCTGCCCCCTCAGCCCGAAATGCCCCCTACTTCCCTTGTGACAAGGCCAGCAAAGAAGAAGAGAGACCCTCCTCGGAAGATGAGATTGCCCTGCATTTCGAGCCCCCCAATGCACCCCTGAACCGGAAGGGTCTGGTTAGTCCACAGAGCCCCCAGAAATCTGACTGCCAGCCCAACTCGCCCACAGAGTCCTGCAGCAGT |
| After | ATGGCGAGCCCGGCGGACAGCTGCATCCAGTTCACCCGTCACGCGAGCGATGTGCTGCTGAACCTGAACCGTCTGCGTAGCCGTGACATCCTGACCGATGTGGTTATTGTGGTTAGCCGTGAACAGTTTCGTGCGCACAAGACCGTGCTGATGGCGTGCAGCGGTCTGTTCTACAGCATTTTTACCGACCAACTGAAATGCAACCTGAGCGTTATCAACCTGGACCCGGAAATTAACCCGGAGGGTTTCTGCATCCTGCTGGACTTTATGTACACCAGCCGTCTGAACCTGCGTGAAGGCAACATTATGGCGGTGATGGCGACCGCGATGTACCTGCAAATGGAGCACGTGGTTGATACCTGCCGTAAGTTCATCAAAGCGAGCGAAGCGGAGATGGTTAGCGCGATTAAGCCGCCGCGTGAGGAATTTCTGAACAGCCGTATGCTGATGCCGCAAGACATCATGGCGTACCGTGGCCGTGAAGTGGTTGAGAACAACCTGCCGCTGCGTAGCGCGCCGGGTTGCGAAAGCCGTGCGTTCGCGCCGAGCCTGTATAGCGGTCTGAGCACCCCGCCGGCGAGCTACAGCATGTATAGCCACCTGCCGGTGAGCAGCCTGCTGTTCAGCGACGAGGAATTTCGTGATGTGCGTATGCCGGTTGCGAACCCGTTTCCGAAAGAGCGTGCGCTGCCGTGCGACAGCGCGCGTCCGGTTCCGGGTGAATACAGCCGTCCGACCCTGGAAGTGAGCCCGAACGTTTGCCACAGCAACATCTACAGCCCGAAGGAAACCATTCCGGAGGAAGCGCGTAGCGATATGCACTATAGCGTGGCGGAGGGTCTGAAACCGGCTGCGCCGAGCGCGCGTAACGCGCCGTATTTCCCGTGCGACAAGGCGAGCAAAGAGGAAGAGCGTCCGAGCAGCGAAGATGAGATTGCGCTGCACTTTGAACCGCCGAACGCGCCGCTGAACCGTAAGGGTCTGGTTAGCCCGCAGAGCCCGCAGAAGAGCGATTGCCAACCGAACAGCCCGACCGAGAGCTGTAGCAGC |
