# Supplementary material for: Generation and characterization of a monoclonal antibody against human BCL6 for immunohistochemical diagnosis
Source: PLoS One. 2019 May 7;14(5):e0216470. doi: 10.1371/journal.pone.0216470 (PMC6504089; doi:10.1371/journal.pone.0216470)
Supplement: S1 Fig — (DOC) [file pone.0216470.s002.doc]

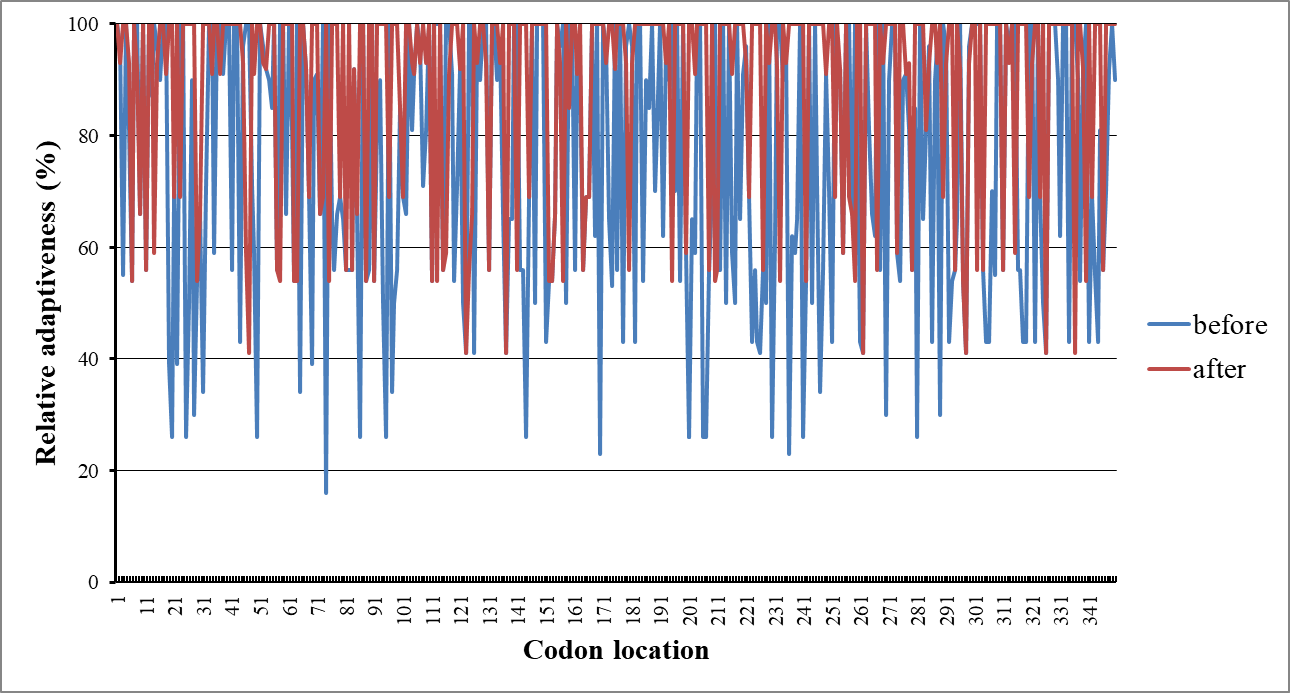


**S1 Fig. Comparison of BCL6_1-350_ Relative adaptiveness between original (before) and optimized sequence (after).** The relative adaptiveness of BCL6_1-350_ coding sequence for *E.coli* was analysed by graphical codon usage analyser (http://gcua.schoedl.de). Then the relative adaptiveness of original and optimized sequences were compared.
